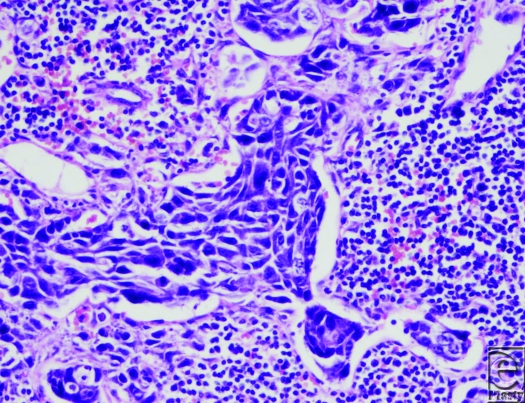# Metastatic Basal Cell Carcinoma: A Case Report and Review of the Literature

**Published:** 2011-04-29

**Authors:** Anthony Vu, Donald Laub

**Affiliations:** University of Vermont College of Medicine, Burlington Vermont

## DESCRIPTION

A 52-year-old patient had basal cell carcinoma diagnosed by biopsy on the left side of his nose. In lieu of definitive surgery to remove the tumor, the patient elected to use a topical herbal treatment with *Sanguinaria candensis* (bloodroot). While this led to an apparent clinical resolution of the cancer, the patient returned 11 years later with deep-seated disease in the same region, which also involved the maxilla. The lesion was removed by a total rhinectomy and partial maxillectomy; reconstruction was achieved with a septal mucosal flap and vertical paramedian forehead flap.

One year subsequently, the patient presented with submandibular lymphadenopathy. A modified radical neck lymphadenectomy was performed and cervical lymph nodes were positive for metastatic basaloid carcinoma. Despite treatment with adjuvant systemic chemotherapy, the patient developed rapid distant bony metastasis and died shortly after.

## QUESTIONS

1. **What is the metastatic risk of basal cell carcinoma?**
2. **What are the treatment options for metastatic basal cell carcinoma?**
3. **How may self-treatment of skin malignancy place the patient at risk for metastatic spread of disease?**

## DISCUSSION

Basal cell carcinoma is the most common skin malignancy, accounting for up to 80% of all cancers arising from the epidermis.^1^ Basal cell carcinoma affects approximately 1 million Americans each year, more than squamous cell carcinoma and melanoma combined.^2^ Surgical excision has long been considered the gold standard of treatment. Surgical excision is generally curative with 5-year cure rates of more than 99% for primary tumors not involving the head.^3^ For lesions involving the head, the 5-year cure rate is 97% for lesions less than 6 mm and 92% for lesions greater than 6 mm. Other surgical options include Mohs micrographic surgery, curettage and electrodessication, and cryosurgery. In a Cochrane review of different treatment modalities, one study showed no significant difference between Mohs micrographic surgery and surgical excision in recurrence rates at 30 months for high-risk facial basal cell carcinomas.^4^

While the lifetime risk of basal cell carcinoma is high, it is well known to physicians that metastasis is relatively rare. Using the criteria proposed by Lattes and Kessler^5^ in 1951, studies have indexed a metastasis rate of 0.0028% to 0.5%.^6^ Since 1894, there have been around 300 reported cases.^7^ Wadhera et al^6^ felt the currently published rate of metastasis underestimated the metastatic risk. In a review by Randle,^8^ tumors with any of the following characteristics should be considered high-risk for metastatic potential: long duration, location in the mid face or ear, diameter larger than 2 cm, aggressive histological subtype, previous treatment, neglected, or history of radiation.^8^ There is a 2% incidence of metastasis for tumors larger than 3 cm in diameter. The incidence increases to 25% for tumors larger than 5 cm in diameter and 50% for tumors larger than 10 cm in diameter.^9^ Increased tissue invasion and extension of the tumor into adjacent anatomical structures also enhance metastatic potential.^9^ Immunosuppression and evidence of perineural spread or invasion of blood vessels have also been implicated as risk factors for metastasis.^10^

For patients with metastatic disease, morbidity and mortality remain exceedingly high. The biggest risk factors for metastasis are tumor size, depth, and recurrence, despite optimal treatment. Primary basal cell carcinoma metastasizes usually via lymphatics, although it also spreads hematogenously. Metastasis most commonly occurs in regional lymph nodes, lung, and bone although there have been documented cases involving the spinal cord,^11^ parotid gland,^12^ skin,^13^ bone marrow, spleen, liver, adrenal glands,^14^ brain, dura mater, esophagus, heart, and kidney.^15^ The prognosis for these patients is poor with a mean survival time of only 8 months from the time at diagnosis.^16^ In cases where metastasis is only to lymph nodes, patients live up to an average of 3.6 years.^17^ There has been one reported case in which a patient lived 25 years after diagnosis.^18^ Median age at the first sign of metastasis is 59 years, while the median interval between the onset of the primary tumor and the first sign of metastasis is 9 years.^6^

There are currently no established guidelines for the treatment of metastatic disease namely because all forms of treatment thus far have provided dismal results. Systemic chemotherapy has been attempted with mixed results. Combinations of 5-fluorouracil, bleomycin, and methotrexate have been unsuccessful thus far.^19^,^20^ However, there has been one case with a positive response to cyclophosphamide and *cis*-diamine dichloroplatinum in a patient with pulmonary metastasis.^21^ Cisplatin-based therapy for patients with evidence of metastasis has also been shown to be of some benefit.^22^^-^24

The incidence of basal cell carcinoma will continue to increase over the years as the baby-boomer generation continues to age. In some cases, patients will try to self-treat the tumor with alternative medicine, such as our patient who used bloodroot.^25^ With the advent of the Internet and an unregulated herbal therapeutic industry, patients will continue to present physicians after the use of these anecdotally supported treatments.^26^ We discourage the use of these forms of treatment, especially within the current culture of practicing evidence-based medicine. Plastic surgeons need to be aware of the poor prognosis that metastatic basal cell carcinoma carries. Because adequate treatments are not available for metastasis, prevention should be practiced by all providers through vigilant monitoring of suspicious skin lesions and early surgical excision of primary tumors.

## Figures and Tables

**Figure:**